# Direct RNA targeted in situ sequencing for transcriptomic profiling in tissue

**DOI:** 10.1038/s41598-022-11534-9

**Published:** 2022-05-13

**Authors:** Hower Lee, Sergio Marco Salas, Daniel Gyllborg, Mats Nilsson

**Affiliations:** Science for Life Laboratory, Department of Biochemistry and Biophysics, Stockholm University, 171 65, Solna, Sweden

**Keywords:** Biotechnology, Genomics, Transcriptomics, Biological techniques, Gene expression analysis, Fluorescence in situ hybridization

## Abstract

Highly multiplexed spatial mapping of transcripts within tissues allows for investigation of the transcriptomic and cellular diversity of mammalian organs previously unseen. Here we explore a direct RNA (dRNA) detection approach incorporating the use of padlock probes and rolling circle amplification in combination with hybridization-based in situ sequencing chemistry. We benchmark a High Sensitivity Library Preparation Kit from CARTANA that circumvents the reverse transcription needed for cDNA-based in situ sequencing (ISS) via direct RNA detection. We found a fivefold increase in transcript detection efficiency when compared to cDNA-based ISS and also validated its multiplexing capability by targeting a curated panel of 50 genes from previous publications on mouse brain sections, leading to additional data interpretation such as de novo cell clustering. With this increased efficiency, we also found to maintain specificity, multiplexing capabilities and ease of implementation. Overall, the dRNA chemistry shows significant improvements in target detection efficiency, closing the gap to other fluorescent in situ hybridization-based technologies and opens up possibilities to explore new biological questions previously not possible with cDNA-based ISS.

## Introduction

There is a wide array of technologies for in situ visualization of transcripts having various benefits and drawbacks^1–3^, with many current methods requiring specialized microscopes to resolve diffraction limited spots^4–7^. Although many fluorescent in situ hybridization (FISH)-based methods can have high detection efficiency and/or high multiplexing capability, they have a tradeoff in throughput of limited imaging area^4–6^. Wrangling of data sets covering large areas, large patient cohort studies, or many samples from model organisms needs to be considered, as they are essential to gain biologically relevant knowledge where individual small samples don’t suffice as seen in projects such as the Human Cell Atlas^8^.

Our lab has developed in situ sequencing (ISS) for multiplexed transcript detection within tissue. ISS is based on hybridization of barcoded padlock probes (PLPs) to specific targets before being ligated and amplified by rolling circle amplification (RCA), forming single stranded DNA repeats known as rolling circle products (RCPs)^9,10^. In the latest iteration of ISS, hybridization-based in situ sequencing (HybISS)^10^, RCPs contain barcodes that can be combinatorially decoded by hybridizing primary bridge probes and fluorescently labelled oligonucleotides over multiple cycles and visualized using conventional widefield fluorescence microscopes (Supplementary Fig. 1a). Although ISS has good signal detection and throughput, it suffers from low transcript detection efficiency^9,11^ that can be attributed to the inefficiency of early steps, including cDNA synthesis, PLP hybridization and ligation. By probing mRNA directly in situ, the reverse transcription step can be circumvented and detection efficiency potentially be increased. Our lab has previously explored RNA templated DNA detection using different commercially available DNA and RNA ligases with some success, but generally DNA ligation on RNA substrates have shown higher tolerance for ligation of mismatched substrates^12^, and consequently worse specificity compared to cDNA templated DNA ligation. Here we evaluate an ISS kit using a direct RNA (dRNA) probing chemistry in situ that retains the fundamental benefits of cDNA-based ISS technology.

Combined with sequencing-by-hybridization detection chemistry of HybISS^10^, we applied a targeted probe panel on mouse coronal brain sections for a comparative analysis of methods and demonstration of its capabilities and potential. We show a fivefold increase in transcript detection efficiency compared to cDNA-based HybISS, which allows for additional data interpretation not previously possible such as de novo cell typing. In addition to the increased detection efficiency with the dRNA chemistry, the specificity, multiplexing capabilities and ease of implementation of ISS are maintained. Overall, a dRNA-based ISS approach expands the analytical capabilities by closing the gap to other FISH-based methods while maintaining a high level of multiplexing and throughput.

## Results

**Figure 1:**
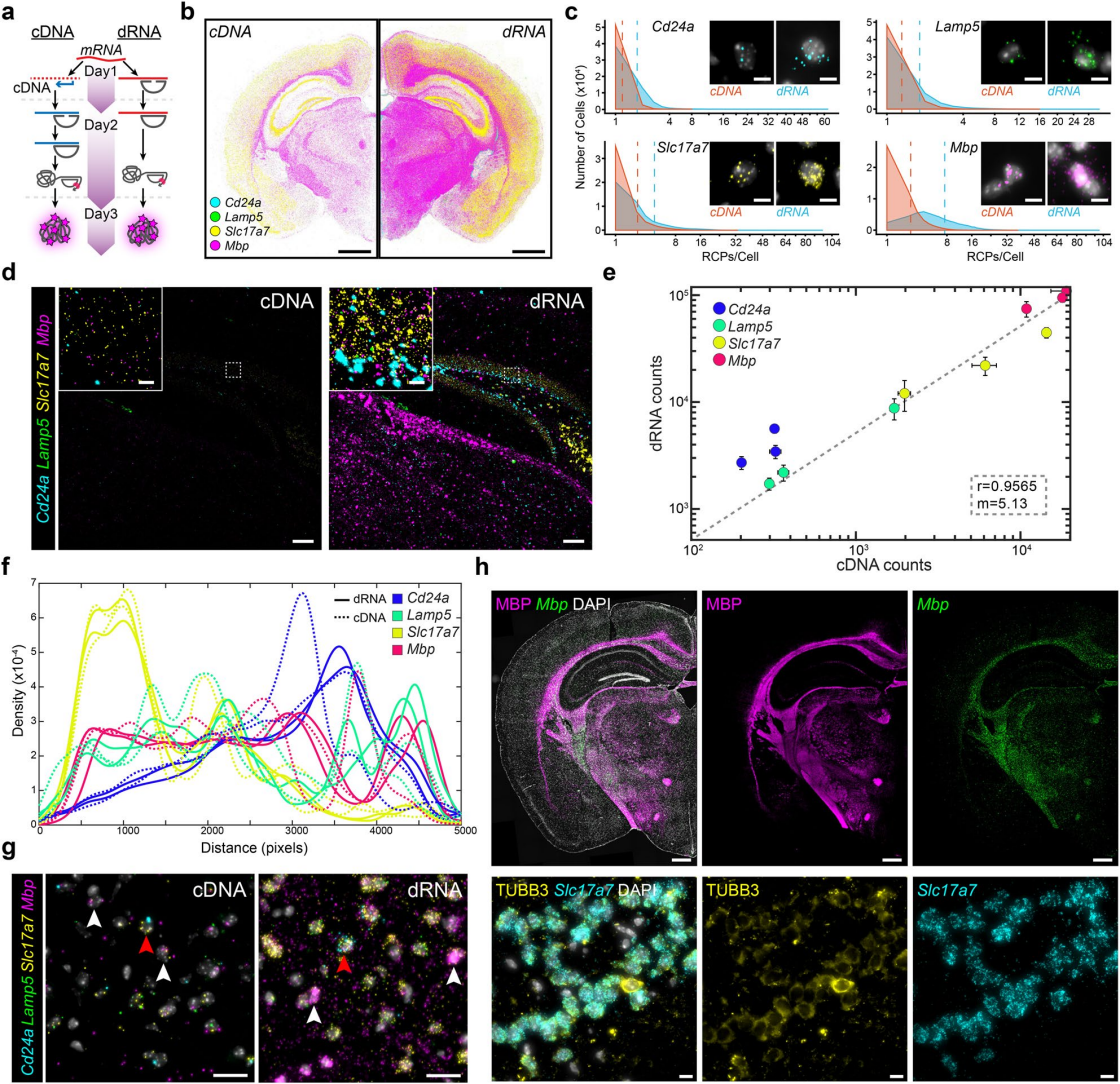
dRNA-HybISS targeting in situ using a 4-plex gene panel. (**a**) Schematic of benchmarking experiment to compare cDNA- and dRNA-based HybISS. (**b**) Expression distribution of 4-plex gene panel (*Cd24a*/*Lamp5*/*Slc17a7*/*Mbp*) across sequential half coronal mouse brain section. Displayed as output from MATLAB analysis pipeline. Scale bar, 1 mm. (**c**) RCP counts per cell of half coronal section and their frequency for each gene in the 4-plex panel. Representative raw images of single cells. Dashed lines represent the mean RCPs/Cell count for the respective chemistries. Scale bar, 5 µm. (**d**) Representative raw image of 4-plex from one of three ROIs (Supplementary Fig. 1e,f). Experiments run in parallel and same post processing intensity level adjustments. ROIs include regions of cortex, hippocampus, and lateral ventricle. Scale bar, 100 µm, inset 10 µm. **e,** Correlation plot of total RCP counts of dRNA against cDNA in three ROIs. X-axis represents cDNA and y-axis dRNA. Each spot of same color represents the 3 ROIs and deviation calculated from consecutive sections. (**f**) Spatial distribution of 4-plex genes across mouse cortex ROI between cDNA and dRNA. (**g**) Multiplexed distribution of 4-plex genes in cortical region. *Cd24a* + cells indicated by red arrowheads, *Mbp* + cells indicated by white arrowheads. Scale bar, 20 µm. (**h**) Colocalization of fluorescent immunohistochemistry with dRNA HybISS. Top panels show MBP protein detection with *Mbp* dRNA-HybISS. Bottom panels show pan-neuronal marker TUBB3 with excitatory neuron marker *Slc17a7*. Scale bar, top 500 µm, bottom 10 µm.

*Increased targeting efficiency and retained specificity of dRNA-ISS.* In the dRNA chemistry from the HS Library Preparation Kit, gene specific PLPs hybridize directly to the mRNA target, before they are ligated, amplified by RCA and fluorescently labelled for detection (Fig. 1a). In order to make use of HybISS detection chemistry^10^, PLP backbone sequences were customized to contain 20 nucleotide (nt) long unique ID sequences that are assigned to each gene of interest to be decoded in a combinatorial manner by first binding ID sequence specific bridge probes that are then used to bind fluorophore conjugated detection oligonucleotides (DO) (Supplementary Fig. 1a and Supplementary Table 1–3).

We first compared dRNA-HybISS to cDNA-HybISS by targeting four genes (i.e. 4-plex) selected for their specificity in marking different cell types including ependymal (*Cd24a*), oligodendrocytes (*Mbp*), and excitatory neurons (*Lamp5* and *Slc17a7*) in a mouse brain coronal section (Fig. 1a,b and Supplementary Fig. 1b). For this comparison, the same number of probes per gene for both dRNA and cDNA were designed to target complementary transcript sequences (Supplementary Table 1). The four genes can be discriminated from each other in a single cycle, but the possibility of combinatorial decoding is still feasible (Supplementary Fig. 1c). To get an overall impression of the increased efficiency of dRNA, the total number of RCPs detected per segmented cell in the sections showed an overall increase in number and frequency for all four genes (Fig. 1c). This is visually clear when comparing images of single cells expressing the various genes (Fig. 1c insets, Supplementary Fig. 1d). Furthermore, we selected three regions of interest (ROIs) encompassing the cortex, hippocampus and lateral ventricle for more detailed analysis (Supplementary Fig. 1e). Comparable images of the ROIs showed clear increased detection efficiency where sub-regional localization of detection could be seen with various densities in the dRNA condition, clear *Mbp* abundance in the corpus callosum, *Cd24a* surrounding the ventricle and *Slc17a7* within the cortex (Fig. 1d and Supplementary Fig. 1f). Total sum of RCPs for each gene was quantified in the ROIs in replicate sections for each condition and found a correlation with a slope of 5.13, indicating an over fivefold increase in detection efficiency using the dRNA approach (Fig. 1e).

Next, we investigated if the signal strength obtained by RCA could vary between the two chemistries by comparing the fluorescence intensities of RCPs formed by dRNA and cDNA-HybISS. The results showed variable intensity depending on the fluorophore (Supplementary Fig. 1g,h), but overall, the signal-to-noise ratio (SNR) of dRNA-HybISS were comparable to cDNA-HybISS, showing that RCA signal amplification efficiency is similar for both probing chemistries (Supplementary Fig. 1i,j).

The increased number of detection events by dRNA-HybISS compared to cDNA-HybISS could in principle be due to increased detection efficiency or as a consequence of off-target detection events, or a combination thereof. To evaluate this, we looked into the spatial distribution of the four targeted genes. Due to the architectural organization of the cortex, we were able to assess the spatial distribution of the four genes along the cortex, observing similar distribution pattern between dRNA and cDNA (Fig. 1f). We further looked into ROIs to observe the spatial distribution of the four genes in more detail. Although cells expressing certain genes could be found within the cortex with both methods (Fig. 1g), it was visually more pronounced in the dRNA approach as indicated by *Mbp* (white arrowheads) and *Cd24a* (red arrowhead) expressing cells. With the increased detection efficiency with the dRNA approach, a much clearer delineation of *Cd24a*^+^ cells lining the ventricle can be observed within the lateral ventricle (Supplementary Fig. 2a, top), as compared to the cDNA method. Furthermore, within the hippocampal formation, a clear separation of *Cd24a*^+^ and *Slc17a7*^+^ cells could be seen that was almost indistinguishable in the cDNA approach (Supplementary Fig. 2a, bottom). Co-localization of protein detection and RNA expression by performing immunohistochemistry (IHC) alongside dRNA-HybISS revealed near identical staining pattern and density across the mouse brain tissue section when comparing Mbp (Fig. 1h, top). When targeting *Slc17a7* together with pan-neuronal marker Tubulin Beta-III (TUBB3) antibody (Fig. 1h, bottom), *Slc17a7* expression co-localized with most of the cells detected with TUBB3 within an ROI expressing *Slc17a7*^+^ cells and no RCPs were observed in cells that were not TUBB3 detected. Comparing the 4-plex dRNA approach to the Allen Mouse Brain Atlas in a cortical ROI, we see overlapping distribution of expression of all genes (Supplementary Fig. 2b). This also applied to other regions as well as overall distribution of expression in the entire coronal section (Supplementary Fig. 2c).

To evaluate unspecific binding of the dRNA probes, we switched the 4-plex probe sets for the different experimental setups, with dRNA probes paired with cDNA chemistry and vice versa. An additional set of cDNA reference probes (*Actb*, *Gapdh*, *Pgk1*, and *Polr2a*) were added as a positive control into the mix of dRNA probes processed through the cDNA protocol (Supplementary Fig. 3a). As expected, no signal was observed in either condition, but only after stripping and labelling with bridge probes for the reference genes, RCPs could be visualized (Supplementary Fig. 3b). A competitive assay using primers of varying concentration targeting the *Mbp* binding sites for the PLPs (Supplementary Fig. 3c) resulted in almost a complete suppression of detectable *Mbp* RCPs (Supplementary Fig. 3d,e). To illustrate the inefficiency of reverse transcription (RT) for cDNA synthesis in situ, we performed an RT step prior to hybridizing the 4-plex panel of dRNA probes before ligation and RCA (Supplementary Fig. 3f). The number of RCPs detected was around 25–35% of the control, indicating incomplete cDNA synthesis and supports the hypothesis that RT is one of the limiting factors of cDNA-ISS, but probably not the only one (Supplementary Fig. 3g,h). Another limiting factor could be the PFA fixation of the cDNA post RT.

**Figure 2:**
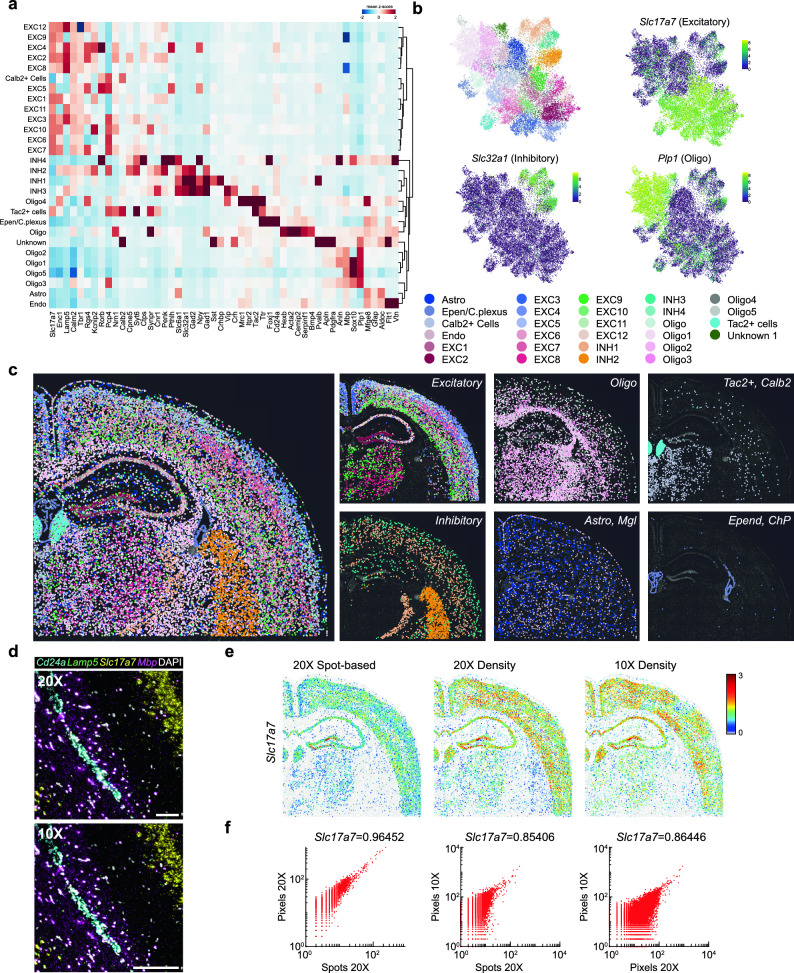
De novo clustering of 50 gene expression in mouse brain coronal section. (**a**) Expression matrix of 50 targeted genes across annotated cell clusters in segmented cells of the imaged region. (**b**) Uniform Manifold Approximation and Projection (UMAP) with de novo cell clustering based on the expression profile of the 50-plex gene panel. Three genes highlighted for their expression to mark pan-excitatory neurons (*Slc17a7*), inhibitory neurons (*Slc32a1*), and oligodendrocytes (*Plp1*). (**c**) Cell-type map across mouse coronal section, highlighting some classes in right panels. (**d**) Raw image comparison of 20X and 10X objective imaging. 200 pixel scale bar, 20X = 64.2 µm, 10X = 128.4 µm. (**e**) 20X objective spot-based detection converted to density-based detection compared to 10X objective density-base detection for *Slc17a7*. (**f**) Correlation comparison of 20X spot- and density-based detection to 10X-density based detection.

*Multiplexing capacity of dRNA-HybISS for *de novo* cell typing in mouse brain sections.* To test the application and potential of the dRNA-HybISS, we targeted a panel of 50 genes (50-plex) curated based on previous publications to map cortical and hippocampal cell types: 33 genes from *Codeluppi et al.*^6^ and 17 from *Qian et al.*^11^ (Supplementary Fig. 4a, and Supplementary Table 1). Targets were probed sequentially over 14 rounds and then merged to create a composite image (Supplementary Fig. 4b-f). The expression map obtained was then segmented to cells based on nuclear DAPI staining. Due to the increased RCP count per cell, we were able to perform de novo clustering on the data to resolve 28 clusters using leiden algorithm, which would not be possible with cDNA-HybISS (Fig. 2a,b and Supplementary Note 1). While most of the neuronal cell types do not present unique markers in the panel, non-neuronal clusters were easily characterized by the expression of cell type-specific markers. Since both excitatory and inhibitory clusters had a similar expression pattern between them, difficulties for discriminating analogous cell types within excitatory and inhibitory cells were found. However, upon further examination of the excitatory clusters, clear gene expression profiles as well as distinct spatial profiles could be seen across the majority of the subclusters (Supplementary Fig. 5a-c). These cell clusters can then be mapped back to a spatial position in the tissue for further analysis (Fig. 2c).

Additionally, due to amplified signals allowing for rapid imaging and coupled with the increased detection efficiency with dRNA, imaging at an even lower magnification to further increase throughput could be explored. In parallel, we obtained a dataset with 10X imaging as a proof of concept (Fig. 2d). Here we produced expression maps based on a density threshold, not spots, and assigned them to segmented cells (Fig. 2e and Supplementary Fig. 6a). Indeed, we could produce expression 10X density maps that were well correlated to 20X spot imaging but with higher throughput (Fig. 2f and Supplementary Fig. 6b). To start to explore this at a cell clustering level, we attempted to cluster our 10X dataset to achieve cell type discrimination at a much more superficial level and obtained convincing results that such an approach could be possible (Supplementary Fig. 7a-c). This could also be an alternate strategy for imaging in a sequential manner and a good candidate for compressed sensing^17^.

**Figure 3:**
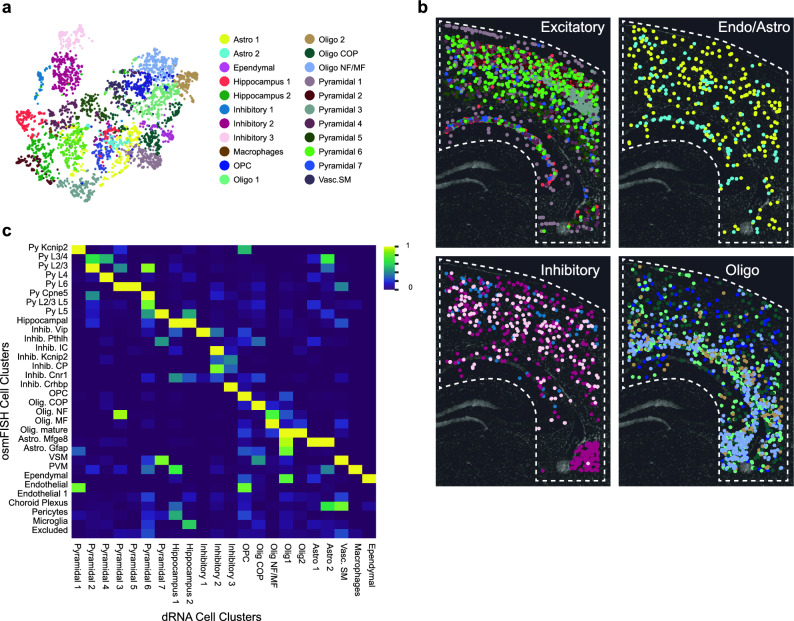
De novo clustering of somatosensory cortex region and comparison to osmFISH cell type clustering. (**a**) UMAP of cell clusters using 33-gene panel within outline ROI. (**b**) Cell-type map of most cell clusters superimposed on DAPI nuclear image. All cell clusters mapped in Supplementary Fig. 8c. (**c**) Similarity map of osmFISH cell clusters compared to dRNA-HybISS de novo clusters obtained by integrating both datasets using Spatial Gene Enrichment (SpaGE)^25^. Color bar corresponds to normalized similarity between clusters detected in both datasets.

*Comparison to published osmFISH dataset.* In order to evaluate how well the unsupervised clustering works with the dRNA method, we compared the dataset acquired to that published by *Codeluppi *et al. implementing osmFISH^6^. We cropped our dataset to a similar region of the somatosensory cortex, CA1 of the hippocampal formation, and lateral ventricle. We then re-clustered our dataset using the same 33 genes with the aim of obtaining comparable clusters. In this region, we defined 22 clusters compared to the 32 clusters found in the osmFISH dataset (Fig. 3a and Supplementary Fig. 8a,b), that was spatially mapped back onto the tissue (Fig. 3b and Supplementary Fig. 8c). Similarity in expression between different clusters shows high correspondence for most of the clusters found with the two techniques (Fig. 3c). The comparison of the clusters found by both in situ methods and published single-cell RNA-sequencing (scRNA-seq)^18^ shows that, despite having lower detection efficiency, dRNA-HybISS is able to define cell types with a similar resolution level as osmFISH (Supplementary Fig. 9a,b). A more elaborate description of this comparison is presented in Supplementary Note 2.

Additionally, when comparing throughput of the two methods, osmFISH imaged an area of 3.96 mm^2^, 9460 fields of view in about 509 min while dRNA-HybISS imaged an area of 52.7 mm^2^, 15,372 fields of view in just over 26 min with 20X and 10X imaging further reduced imaging time to just over 10 min. This substantial amount of time being saved with the increased area throughput is vital in cell atlas projects or to shed insights to biological questions in large tissue samples.

## Discussion

The dRNA-HybISS method presents itself as an improved alternative to the cDNA-based HybISS technology^10^ as it demonstrates a fivefold improvement in transcript detection efficiency, while maintaining specificity, and same degree of throughput and multiplexing capabilities. De novo clustering of ISS data can be performed for the first time where traditionally, clustering of cDNA-based HybISS requires additional scRNA-seq data as a prior. This increase in detection efficiency closes the gap in the analytical capabilities to that of other FISH based techniques as demonstrated by comparing our results to osmFISH^6^.

Currently, alternative protocols that involve the direct probing of RNA with PLPs and RCA, such as SCRINSHOT^19^ and targeted ExSeq^20^ have also exhibited improved transcript detection efficiency. None of these methods have been as extensively benchmarked as in the current study, and the benefits of a commercial kit increases the accessibility of targeted spatially resolved transcriptomic technology to the wider audience in the research community. It takes away the need of in-house technical knowledge of probe design, assay troubleshooting and optimization and the logistical hurdles that are time consuming and inconvenient for labs to adopt a new technology. However, the current lack of “plug-and-play” instrumentation and software solutions remains a hurdle for implementation of this method for many labs.

Briefly investigating the discrepancies in detection efficiencies of both cDNA and dRNA approaches, we found that in situ RT is incomplete. However, the incomplete in situ RT alone does not contribute to the fivefold increase in sensitivity of the dRNA approach. We also speculate that the post fixation step after RT further contributes to the decrease in sensitivity of the cDNA approach. Although the mechanism for this is hard to prove, we believe that the chemical cross-links formed between the cDNA and component of the cellular matrix, with the purpose to retain the cDNA in the fixed cells, may reduce RCA efficiency if it happens within the probe target sequence, since it is difficult for DNA circles to serve as efficient templates for RCA when topological linked^26^. Since the cDNA synthesis in situ is short (must by definition be shorter than the corresponding mRNA), this could constitute a large fraction of all available cDNA molecules.

The dRNA-HybISS method enables more data being generated per cell, and allow detection of mRNAs otherwise being challenging to detect with the cDNA approach, providing opportunities to reach conclusions to a wider range of biological questions. The increased detection efficiency has potential drawback of risk to optical crowding where individual RCPs cannot be distinguished from each other using combinatorial decoding. In order not to confuse the comparison with the cDNA-based method due to crowding, we performed decoding in a non-combinatorial fashion in this work. This is a feasible approach if multiplexing levels are relatively low, such as a 50 gene panel that can be decoded in 14 rounds of imaging, particularly when using 10X imaging. For higher multiplexing, one can group genes for an optimal combinatorial experiment without optical crowding by using prior knowledge from, for example, scRNA-seq data sets. Alternatively, a combination of combinatorial and non-combinatorial decoding cycles can be applied which adds on experimental and imaging rounds, but enables generation of dense, yet not optically crowded data.

Here we also show a proof of concept that an even lower magnification objective (10X) can be used to identify the level of expression of each cell based on signal density to further increase imaging throughput and possibly a good candidate to combine with published compressed sensing strategies^17^ as a solution for optical crowding.

As with all spatial methods, depending on the biological question being asked, the ideal method should be chosen. For example, our current cell-typing pipeline, pciSeq, does not require high detection efficiency per cell to robustly define cell types^11^, making the traditional cDNA-based HybISS sufficient for cell type mapping. However, should one aim to identify low abundant transcripts, dRNA-HybISS would be a better option. Additionally, the dRNA method would be a useful method for FFPE tissues where RNA is more degraded and where other non-amplified FISH methods require well preserved RNA to tile multiple probes across.

To further evaluate dRNA-HybISS, in its multiplex capability and the data quality that it generates, we were able to cluster cell types in a mouse brain section with a panel of 50 genes and compare it to published data sets from both scRNA-seq and osmFISH. From our 50-plex experiment data, we were able to robustly decode 50 genes sequentially to confidently identify cell clusters, which have shown good correlation with both scRNA-seq and osmFISH data set, pointing to the fact that the HybISS chemistry is very much compatible with highly multiplexed experiments and also generates high quality data. To scale up beyond 50 genes in a combinatorial manner, decoding strategies have to be implemented to be able to resolve densely packed spots. With well-planned decoding, increasing number of cyclic rounds and implementing automation, targeting hundreds of genes is possible to answer most biological questions in a cost-effective manner. The full potential of dRNA-HybISS has yet to be explored, but nonetheless, we believe that dRNA-HybISS can be a powerful tool for cell typing especially when combined with scRNA-seq data for gene target selection.

## Methods

### Probe selection and design

Genes were selected based on previous publications to delineate cell types in adult mouse brain sections. Subsets of the 50-plex panel were taken from^6^ and^11^. The 4-plex panel is contained within the 50-plex panel. Gene lists were sent to CARTANA with accompanying customized ID sequences for in-house HybISS chemistry detection. For the 4-plex gene assay, probes were designed by CARTANA to target matching complimentary mRNA and cDNA sequences to suit the different chemistries for benchmarking studies. The same number of probes per specific gene were designed for both dRNA and cDNA approach for a fair comparison. The number of probes used for both dRNA and cDNA were also kept constant at a final concentration of 10 nM per probe. Target sequences and PLP design is CARTANA propriety information and are unknown to users and only targeted exons and number of probes per gene are known (Supplementary Table 1). Mouse reference genes for cDNA-HybISS method were designed as previously published^10^ and sequences can be found in Supplementary Table 1.

### Tissue

Mouse tissue was obtained from the Allen Brain Institute under the SpaceTx consortium. Fresh whole mouse brain tissue was cryopreserved in optimal cutting temperature (OCT) and sectioned with a cryostat (CryoStar™ NX70) at 10 μm and collected on SuperFrost Plus microscope slides. Slides stored at -80 °C were air dried for five minutes before respective protocols were performed.

### cDNA-HybISS protocol

The protocol was followed as published^10^ and at protocols.io (10.17504/protocols.io.xy4fpyw). As with all dRNA probes, cDNA probes for the 4-plex assay were also provided by CARTANA to match complementary sequences of the dRNA target sequences.

### dRNA-HybISS protocol

CARTANA provided reagents in kits (High Sensitivity library preparation kit) with an accompanying protocol that was followed. Briefly, after tissue fixation, dRNA probe mix (Probes + RM1) was incubated on tissue section overnight at 37 °C in hybridization buffer followed by a washing step (WB4) and then incubated in a ligation mix (RM2 + Enzyme 1 & 2) at 37 °C for 2 h. After washes, RCA was performed overnight at 30 °C and labelled for detection. Protocols for tissue fixation, both RCA and detection are exactly the same as with cDNA-HybISS.

### IHC staining protocol

After dRNA-HybISS RNA detection, tissue was blocked with PBTA (PBS, 5% normal donkey serum (Jackson ImmunoResearch), 0.5% Triton-X 100) for one hour. Then sections were incubated with primary antibodies, either MBP (Abcam, ab7349) or TUBB3 (BioLegend, 801,213) overnight at + 4 °C. Sections were then washed three times with PBS and incubated with secondary antibodies (Alexa Fluor anti-rat 488 and anti-mouse 555) for 2 h at room temperature and counterstained with DAPI.

### Imaging

All images were obtained with a Leica DMi8 epifluorescence microscope equipped with an external LED light source (Lumencor® SPECTRA X light engine), automatic multi-slide stage (LMT200-HS), sCMOS camera (Leica DFC9000 GTC), and objectives (HC PL APO 10X/0.45; HC PL APO 20X/0.80; HCX PL APO 40X/1.10 W CORR). Multispectral images were captured with microscope equipped with filter cubes for 6 dye separation and an external filter wheel (DFT51011). Image scanning was performed by outlining ROIs that could be saved for multi-cycle imaging tiled imaging with 10% overlap. Z-stack imaging of 10 µm at 0.5 µm steps to cover the depth of the tissue.

### Image processing

Imaging-data was processed and analyzed with an in-house pipeline based on the programming language MATLAB. All associated software can be found in the repository (https://github.com/Moldia/HybrISS ).

Maximum intensity projection was performed on each field of view in order to obtain a 2-dimensional representation of each tile. Then, stitching of the different tiles was performed using a MATLAB implementation of MIST^22^ algorithm, obtaining, after exporting, different tiff images corresponding to each channel and round. After aligning the images, and top-hat filtering them, signals were identified by manually defining an intensity and size threshold on each channel. For experiments including multiplexing, a spot-associated quality score was calculated by dividing the intensity score of the channel where the signal was detected by the sum of the intensities of all the other channels, excluding DAPI. Assuming a perfect alignment between images, each signal in the 1st cycle was associated with its closer signal in the 2nd cycle generating a possible barcode. A quality score was given to each union, being the distance between signals expressed in number of pixels. For each of these barcodes, a final quality score (Q) was calculated as:

$$Q = \prod\limits_{{i = 1}}^{{n - 1}} {q_{i} q_{{i + 1}} - kd_{{i(i + 1)}} }$$

where n = 2, since 2 cycles have been used in the combinatorial experiment. d_i(i+1)_ represents the distance between two signals in different rounds and is modulated by the parameter k, which can be tuned. The variables q_i_ and q_i+1_ represent the quality of a signal in the first cycle and the second respectively. Barcodes were filtered based on their final quality score ( Q ) , keeping only those multiplexed signals presenting a high quality (Q > 0).

### Cell segmentation

DAPI staining was used to identify cell nuclei by filtering its signal based on a manually set intensity threshold. Watershed segmentation was performed on top of that in order to identify approximate cell boundaries. Signals detected within the cell boundaries of a cell were assigned to it, capturing the expression profiles of individual cells.

### Data analysis

The data analysis was performed using a customized pipeline based on SCANPY^23^. Segmented cells were filtered depending on their gene expression, excluding both lowly expressed genes and cells without too few counts. The expression of each gene was normalized and log-transformed. However, In the case of the density-based data, acquired with 10X objectives, the density of each gene on each cell was divided by the total area of each cell in order to adjust for cell size. Then, Leiden^24^ clustering was performed on the normalized gene/cell matrix and resulting clusters were manually annotated. The representations of the 50-plex shown were based on the plotting functions of SCANPY.

### scRNA-seq integration

Spatial gene enrichment (SpaGE)^25^ was used to integrate both spatial datasets (dRNA-HybISS and osmFISH) with the scRNA-seq dataset from Zeisel et al^18^. Nearest neighbors were used to find the top closest cells from the spatial datasets to each of the cells of the scRNA-seq dataset used. This metric was used to calculate the similarity between the spatial clusters and the single cell clusters.

### Supplementary Information

Supplementary Information.

Supplementary Table 1.

Supplementary Table 2.

Supplementary Table 3.

Supplementary Table 4.

Supplementary Table 5.

Supplementary Table 6.

## Data Availability

The supporting data regarding the findings of this study is openly available at https://zenodo.org/records/6809889. The raw tile images (several terabytes) are available from the corresponding author upon reasonable request.

## Code availability

All code is available online at https://github.com/Moldia/HybrISS.
