# Author Correction: Direct RNA targeted in situ sequencing for transcriptomic profiling in tissue

**DOI:** 10.1038/s41598-024-53557-4

**Published:** 2024-02-07

**Authors:** Hower Lee, Sergio Marco Salas, Daniel Gyllborg, Mats Nilsson

**Affiliations:** Science for Life Laboratory, Department of Biochemistry and Biophysics, Stockholm University, 171 65, Solna, Sweden

Correction to: *Scientific Reports* 10.1038/s41598-022-11534-9, published online 13 May 2022

The original version of this Article contained errors.

In the original version of this Article, the Data availability URL was omitted.

“Data availability

Pre-processed images are available at (Will be available at FigShare repository). The raw tile images (several terabytes) are available from the corresponding author upon reasonable request.”

now reads.

“Data availability

The supporting data regarding the findings of this study is openly available at https://zenodo.org/records/6809889. The raw tile images (several terabytes) are available from the corresponding author upon reasonable request.”

In addition, Supplementary Table 1, which contained information regarding Targeted genes and Probe sequences, has been updated.

The incorrect Supplementary Table 1 appears below.

The original version of this Article has been corrected.

### Supplementary Information

Supplementary Information.

