# Supplementary material for: Direct RNA targeted in situ sequencing for transcriptomic profiling in tissue
Source: Sci Rep. 2022 May 13;12:7976. doi: 10.1038/s41598-022-11534-9 (PMC9106737; doi:10.1038/s41598-022-11534-9)
Supplement: Supplementary file 1 — Supplementary Information. [file 41598_2022_11534_MOESM1_ESM.pdf]

SUPPLEMENTARY INFORMATION

Direct RNA targeted in situ sequencing for transcriptomic profiling in tissue

Hower Lee†, Sergio Marco Salas†, Daniel Gyllborg*, Mats Nilsson*

Science for Life Laboratory, Department of Biochemistry and Biophysics, Stockholm University, 171 65, Solna, Sweden

† These authors contributed equally to this work

** Corresponding authors*

Supplementary Information contains 2 Notes, 9 Figures and 6 Tables

DATA

Supplementary Note 1: Description of the clusters identified in Figure 2
Supplementary Note 2: Comparison of clusters

Supplementary Figure 1: dRNA-HybISS method and comparison to cDNA-HybISS using 4-plex gene panel
Supplementary Figure 2: Validation of dRNA-HybISS specificity using 4-plex gene panel
Supplementary Figure 3: Control experiments of dRNA-HybISS specificity using 4-plex gene panel
Supplementary Figure 4: Multiplex implementation of dRNA-HybISS to target panel of 50 genes
Supplementary Figure 5: Excitatory neuron subcluster diversity
Supplementary Figure 6: 10X imaging analysis of *Mbp/Lamp5/Cd24a*
Supplementary Figure 7: Cell type identification using 10X imaging
Supplementary Figure 8: Subset gene panel and region clustering and comparison to osmFISH
Supplementary Figure 9: Method comparison to single-cell RNA-sequencing defined clusters

Supplementary Table 1: Padlock probes and relevant sequences
Supplementary Table 2: Bridge probe sequences
Supplementary Table 3: Detection oligonucleotide sequences and fluorophores
Supplementary Table 4: 50-plex gene imaging rounds
Supplementary Table 5: 50-plex individual cell expression
Supplementary Table 6: 50-plex main cluster expression

Supplementary Note 1: Description of the clusters identified in Figure 2

A total of 28 clusters were defined after the clustering the segmented data with the Leiden algorithm. Those clusters were annotated based on known cell type markers expressed in different clusters and the direct comparison between these clusters and cell types described by Zeisel *et al*.[1] to be present in the same brain region characterized in this experiment.

A total of 12 clusters were classified as excitatory neurons, characterized by the expression of markers such as *Slc17a7* or *Calb2*. Excitatory neurons were mostly found along the cortex, the hypothalamus and, in less abundance, in the thalamus. Some distinct spatial and molecular diversity could be distinguished within excitatory populations due to the expression markers differentially expressed among different excitatory populations. An example of this diversity are clusters EXC4/5, which are found in Layer3/4 in the cortex and express *Rorb*.

Four different clusters were described to be capturing different inhibitory cell types. Two main groups could be described among the inhibitory clusters: 2 clusters which cells are mainly located in the cortex, and other 2 region-specific clusters, including clusters located in the caudoputamen (INH1) and one cluster in the reticular nucleus of the thalamus (INH2). These two clusters, despite their distinct location in the UMAP (Figure 2b) have been assigned to inhibitory clusters due to the resemblance between their expression profile and the inhibitory clusters' one, although they might need to be classified in a distinct group of cells.

We also described 9 clusters as non-neuronal clusters, since their expression profile presents specific markers for typical non-neuronal cell types. Among these, we found 5 oligodendrocyte-like clusters, characterized by the expression of markers like *Plp1*, *Mbp* or *Sox10*. Most of the cells assigned to these clusters are found in the fiber tracts. We also described one cluster showing an astrocyte-like expression profile, characterized by the expression of markers such as *Gfap* or *Mgfe8*. The population wasn't found in a specific spatial location. We also defined clusters assigned to endothelial cells (1) and ependymal cells and choroid plexus (1).

Some other clusters didn't match well with any specific cell type described in Zeisel *et al*.[1], but were characterized by the expression of certain genes, showing a clear location in the tissue. This is the case of *Tac2*+ cells, located in the medial habenula and *Calb2*+ cells, placed in specific regions of the thalamus. In the case of *Aldoc*+ cells, no specific location was found, but cells were characterized by a high expression of *Aldoc*. Finally, one cluster could not be annotated, since it presents a wide expression across the tissue and their expression profile didn't match any specific cell type, having levels of expression for both neuronal and non-neuronal markers. We believe this cluster could be an artifact due to a bad segmentation of certain cells across the tissue.

Supplementary Note 2: Comparison of clusters

In order to compare the expression of the clusters defined by dRNA-HybISS and osmFISH using the list of 33 genes in Codeluppi *et al*.[3] and the cell types defined using scRNA-seq data from Zeisel *et al*.[1], both datasets were integrated using Spatial Gene Enrichment (Figure 3c).

This integration shows high correspondence for most of the clusters found with the two spatial techniques, even though dRNA-HybISS presents a lower detection efficiency. One-to-one correspondence between clusters was observed for many of the of the non-neuronal cell types, including endothelial cells, perivascular macrophages, vascular smooth muscle cells, pericytes and ependymal cells. Regarding clusters expressing oligodendrocyte markers, we were able to clearly distinguish oligodendrocyte precursor cells (OPC), committed OPCs (Olig COP) and, to some extent newly formed Oligodendrocytes (Olig NF) and myelin forming oligodendrocytes (Olig MF) but further subtypes were not clearly distinguishable.

Within neuronal clusters, some discrepancies were present between the neuronal subtypes. The most important discrepancies between the methods were found among the excitatory cells where both methods showed poor capacities to resolve cell types clearly. In this case, no clear one-to-one correspondence can be found within excitatory clusters, except for Pyramidal L4 and Pyramidal L5, where a correlation between a specific osmFISH and a dRNA-HybISS cluster is observed.

Our clusters were also compared with the cell types described by Zeisel *et al*.[1] by scRNA-seq. As observed when comparing dRNA-HybISS clusters with the osmFISH ones, main cell classes could easily be assigned to each of the clusters detected by dRNA-HybISS (Supplementary Figure 9). Similar conclusions are extracted when comparing the osmFISH dataset with the scRNA-seq based cell types, where osmFISH clusters do not match perfectly the scRNA-seq ones for a considerable amount of excitatory and inhibitory populations.

The comparison of the clusters found by the three methods shows that, despite having lower detection efficiency, dRNA-HybISS is able to define cell types with a similar resolution level as osmFISH. Discrepancies between the clusters defined by scRNA-seq clustering and both spatial methods are consistent, proving the importance of the gene panel curation in targeted methods like these.

Supplementary Figure 1

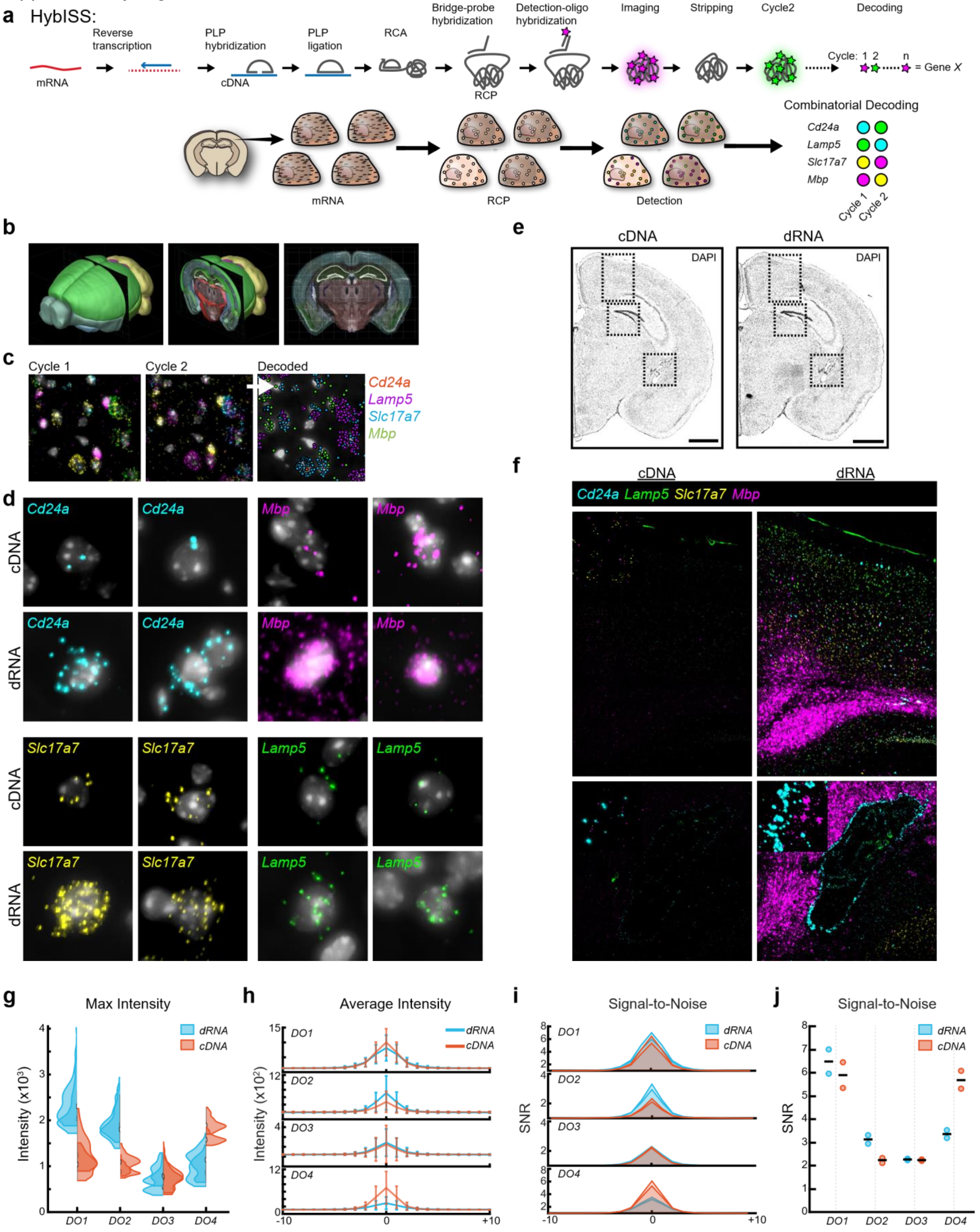

Supplementary Figure 1: dRNA-HybISS method and comparison to cDNA-HybISS using 4-plex gene panel

a, Schematic of HybISS chemistry: (1) Reverse transcription for cDNA synthesis (2) Padlock probe hybridization and ligation (3) Rolling circle amplification (4) Rolling circle product detection and imaging (5) Stripping and repeated cycles for combinatorial barcode decoding.

b, Regional localization of mouse brain coronal section used for analysis. Image credit: Allen Brain Institute.

c, Example of combinatorial decoding that is possible with dRNA-HybISS and gene panels used.

d, Representative images of single cells showing increased detection of individual transcripts in cDNA-HybISS and dRNA-HybISS. Scale bar, 5 µm.

e, DAPI image of half coronal sections for cDNA and dRNA with demarked ROI regions used for analysis.

f, Representative raw images from two of the three ROIs (Supplementary Figure 1e) and the distribution of the 4-plex panel. Experiments run in parallel and same postprocessing intensity signal level adjustments. ROIs include regions of cortex (top) and lateral ventricle (bottom). Scale bar, 100 µm, inset 10 µm.

g, Violin plots displaying max intensity of top 50 RCPs in three ROIs from two samples in each channel measured for cDNA-HybISS and dRNA-HybISS. DO1-AF750, DO2-AF488, DO3-Cy3, DO4-Cy5.

h, Average intensities of measured RCPs in all ROIs (three from each of the two samples for each condition). Pixel intensity measured across a 21-pixel line bisecting RCPs, displayed with standard deviation.

i, SNR measured from (h). Outer 2-pixel measurements at each end of 21-pixel line were used to calculate background noise. Each pixel intensity measurement was divided by the noise to display SNR across the RCPs.

j, Peak SNR from (i) measured across four fluorescent channels.

Supplementary Figure 2

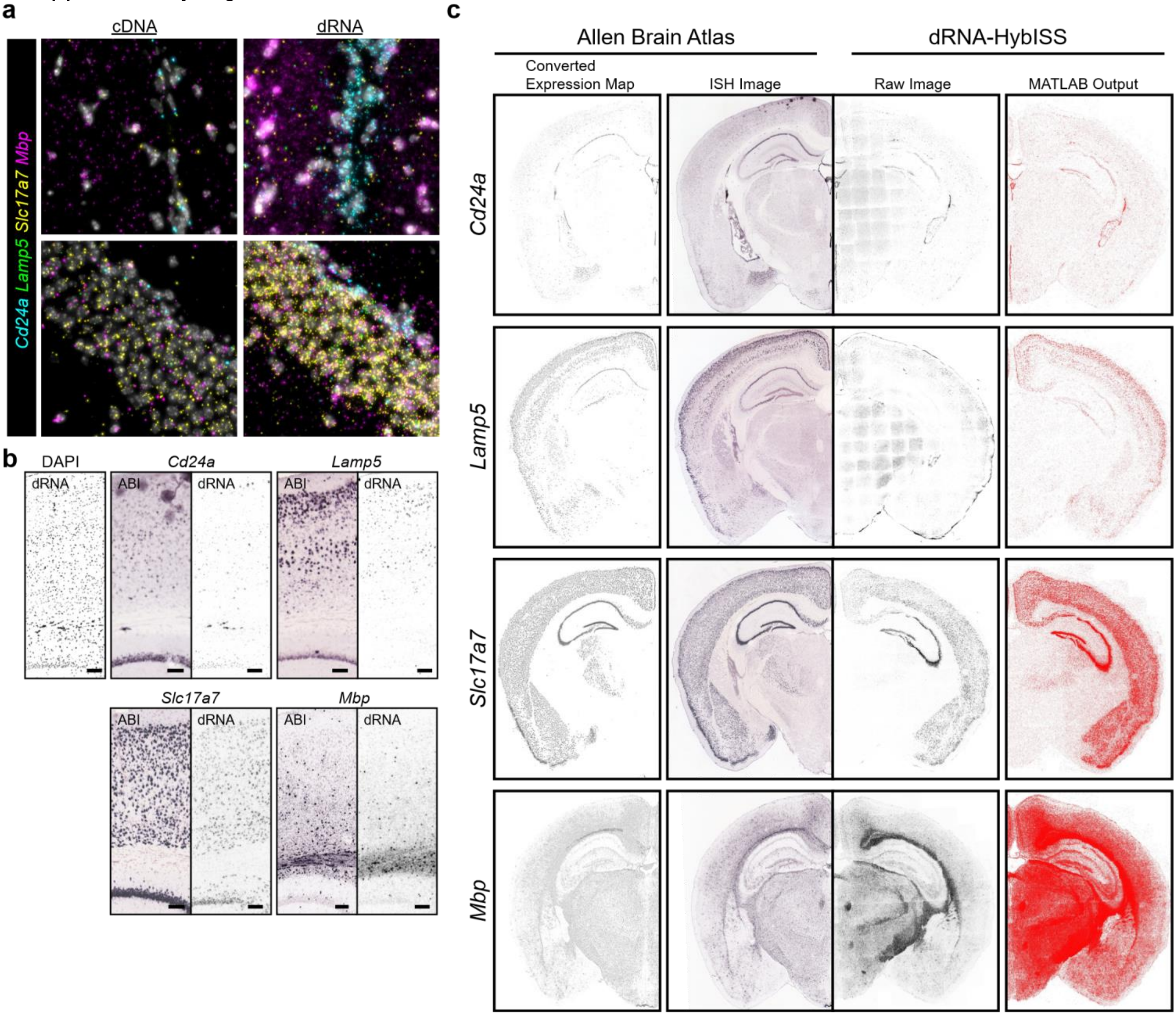

Supplementary Figure 2: Validation of dRNA-HybISS specificity using 4-plex gene panel a, Distribution of 4-plex genes around ventricle (top) and hippocampus (bottom). White arrowhead indicating *Mbp*$^{+}$ cell. Scale bar, 20 µm.

b, Transcript distribution of 4-plex panel compared to Allen Mouse Brain Atlas (ABI) in a region of the cortex. Scale bar, 100 µm. Image credit: Allen Institute.

c, Transcript distribution of *Cd24a/Lamp5/Slc17a7/Mbp* compared to Allen Mouse Brain Atlas[2] in half coronal section. Image credit: Allen Institute.

Supplementary Figure 3

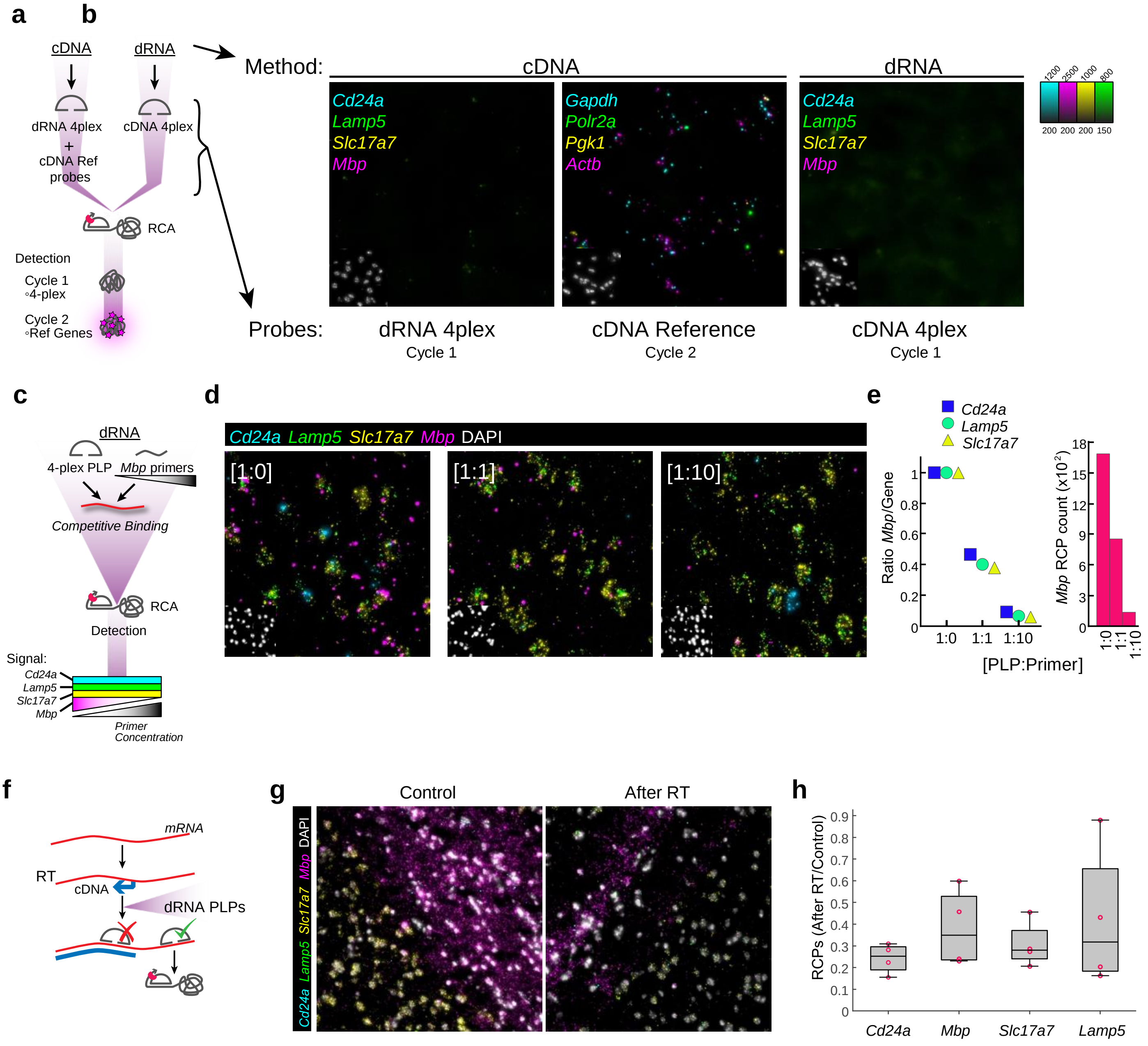

Supplementary Figure 3: Control experiments of dRNA-HybISS specificity using 4-plex gene panel

a, Schematic of negative control experiment where probes for cDNA and dRNA protocols were swapped in each respective protocol. A panel of mouse reference cDNA probes were added into the probe pool for a positive control for cDNA protocol and detected in the second round. **b**, Raw images of experiment in (a) indicating specificity of probes. Inset, DAPI. Scale bar, 10 μm.

c, Experimental schematic of competitive assay with different concentrations of primers that bind to *Mbp* target sequences, and *Cd24a/Slc17a7/Lamp5* as controls. **d**, Images from competitive assay in (c). Scale bar, 10 μm.

e, Calculation showing ratio of *Mbp* to all other genes, normalized to [1:0] ratio (left). Total count of *Mbp* RCPs (right).

f, Experimental schematic for showing inefficiency of cDNA synthesis where reverse transcription is carried out prior to hybridization of dRNA padlock probes. **g**, Images showing dRNA probing after performing cDNA synthesis vs control. Scale bar, 20 μm.

h, Calculation of normalized RCP counts (After RT / Control) from 3 regions of interests (ventricle, cortex and thalamus).

Supplementary Figure 4

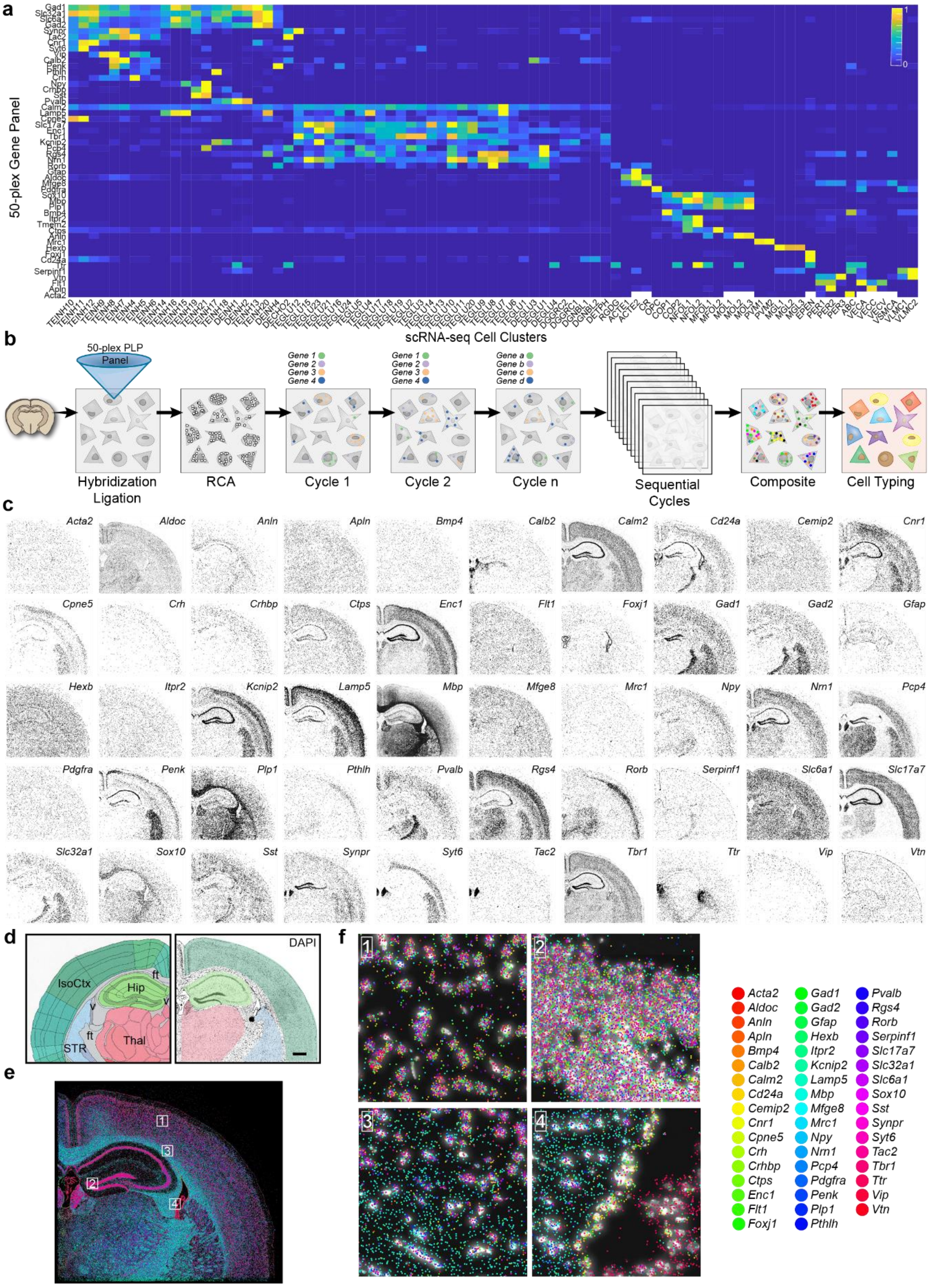

Supplementary Figure 4: Multiplex implementation of dRNA-HybISS to target panel of 50 genes

a, 50-plex gene selection representation in Zeisel et al. scRNA-seq data[1].

b, Schematic overview of experimental workflow of 50-plex gene panel and sequential decoding in order to produce a composite image and cell typing based on transcript expression.

c, Decoded images of all genes in the 50-plex panel imaged over 14 rounds in region of coronal mouse brain.

d, Anatomical position of mouse coronal brain section used in 50-plex experiment (right, area of 0.216 mm^2) and Allen Mouse Brain Reference Atlas (left) covering isocortex, hippocampus, thalamus, striatum, ventricles, and fiber tracts. Scale bar, 500 µm. **e**, 50-plex gene panel, 14 round composite images in coronal mouse brain section. **f**, Regions of interest from (e) showing the transcript density and diversity.

Supplementary Figure 5

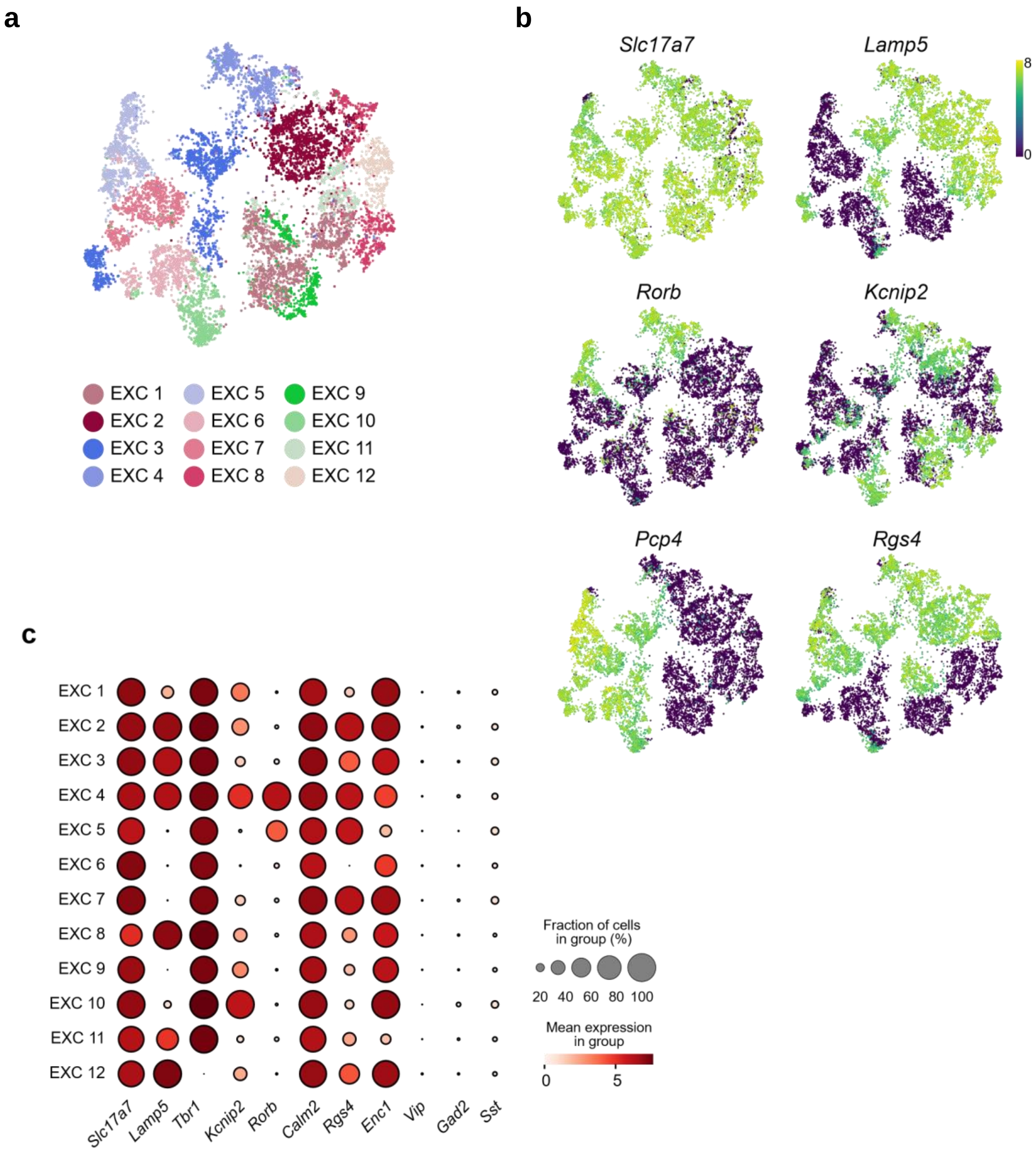

Supplementary Figure 5: Excitatory neuron subclusters

a, UMAP of the excitatory clusters annotated in the 50-plex experiment (Figure 2b). Each cell is colored by the cluster it was assigned to in Figure 2b.

b, UMAP of the excitatory clusters annotated in the 50-plex experiment (Figure 2b). Different UMAPs represent the expression of key marker genes included in the panel that mark the excitatory cells or present different expression depending on the excitatory subtype.

c, Dot plot representing the expression of different genes across excitatory cluster subtypes. Excitatory-specific markers as well as three inhibitory markers (*Vip*, *Gad2* and *Sst*).

Supplementary Figure 6

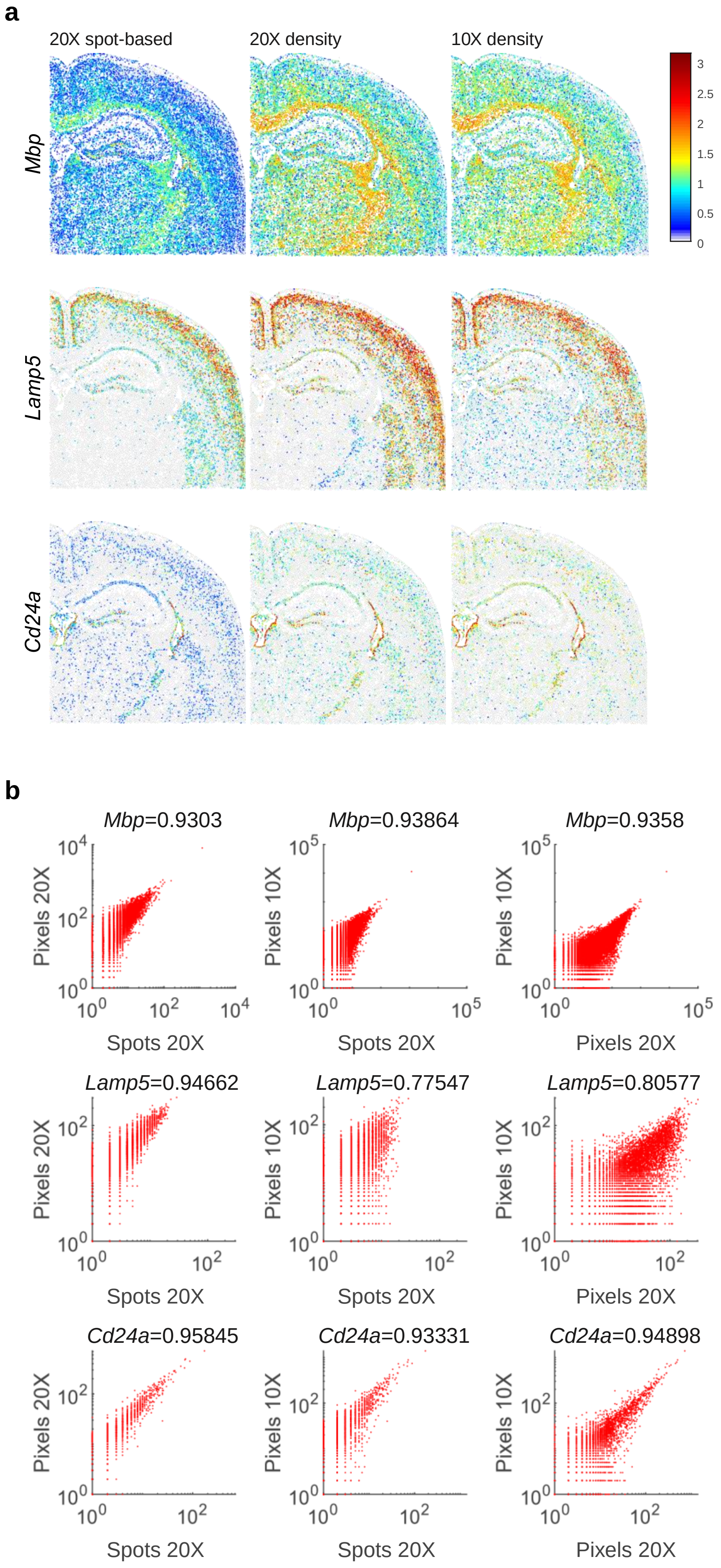

Supplementary Figure 6: 10X imaging analysis of *Mbp/Lamp5/Cd24a*

a, 20X objective spot-based detection along with 20X density-based detection and how it compares to 10X objective density-based transcript detection of *Mbp*, *Lamp5*, *Cd24a*.

b, Expression correlation of pixel and spot detection for 20X objective and same comparison between 10X pixels and 20X spot detection.

Supplementary Figure 7

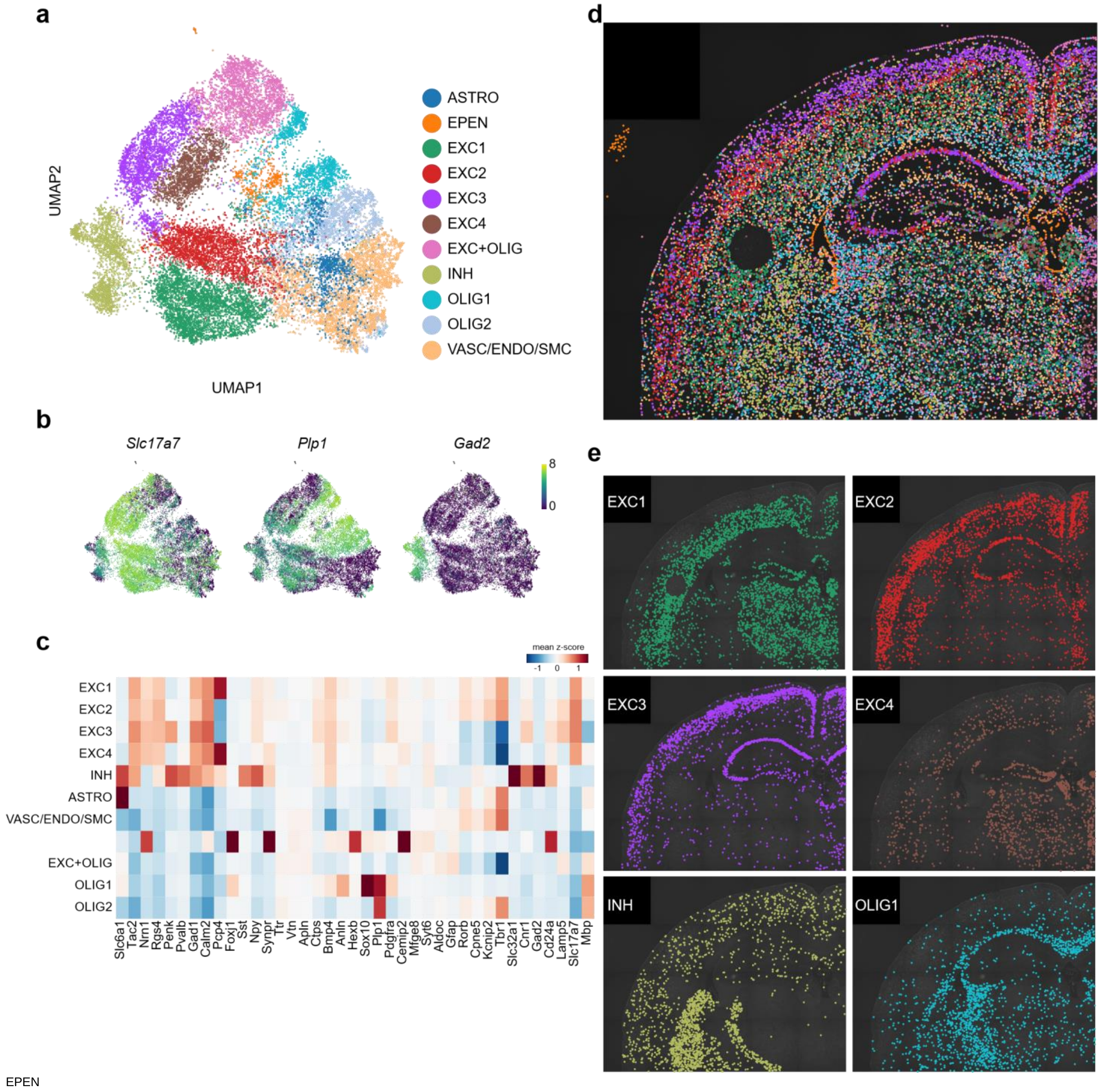

Supplementary Figure 7: Cell type identification using 10X imaging

a, UMAP representing the different clusters identified using density-based expression quantified with 10X images.

b, UMAP representing the expression of *Slc17a7*, *Plp1* and *Gad2* in the different cells defined using density-based expression

c, Heat map representing the mean gene expression of the cells assigned to each of the clusters defined.

d, Spatial distribution of the clusters detected in (a), mapped back on the tissue. Colors correspond to cluster names in (a). **e**, Cell type distribution of specific clusters detected (a) when mapped back on the tissue.

Supplementary Figure 8

a

b

c

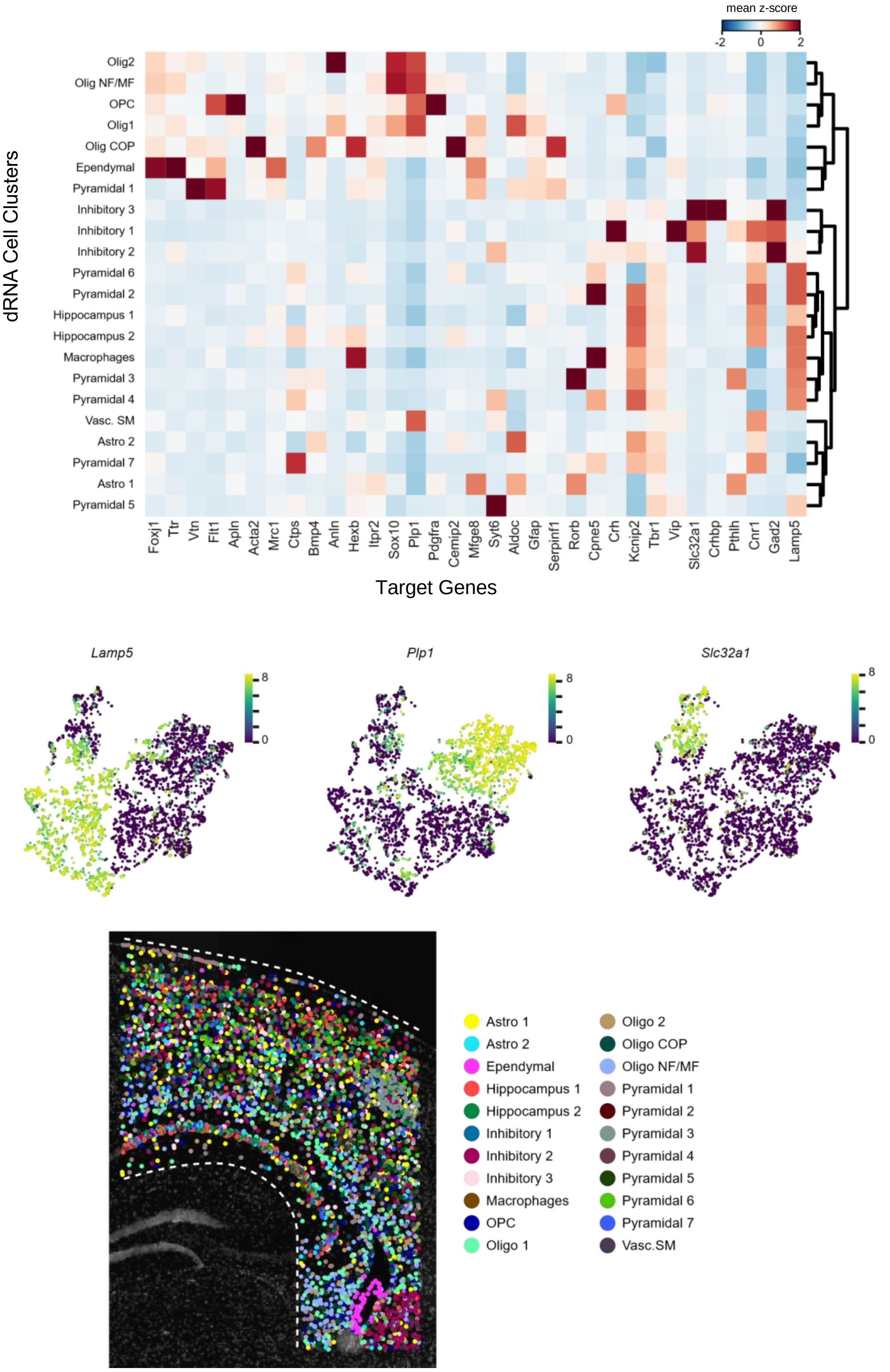

Supplementary Figure 8: Subset gene panel and region clustering and comparison to osmFISH

a, Heat map representing the mean expression of each of the clusters identified by *de novo* clustering of spatial data based on segmented ROI and 33-gene subset taken from the osmFISH panel[1], presented in Figure 3a.

b, UMAP representing the expression of *Lamp5*, *Plp1* and *Slc32a1* in segmented ROI and 33-gene subset.

c, Spatial distribution of all the clusters identified in Figure 3a when mapped back into the tissue. Colors correspond to the ones used in Figure 3a

Supplementary Figure 9

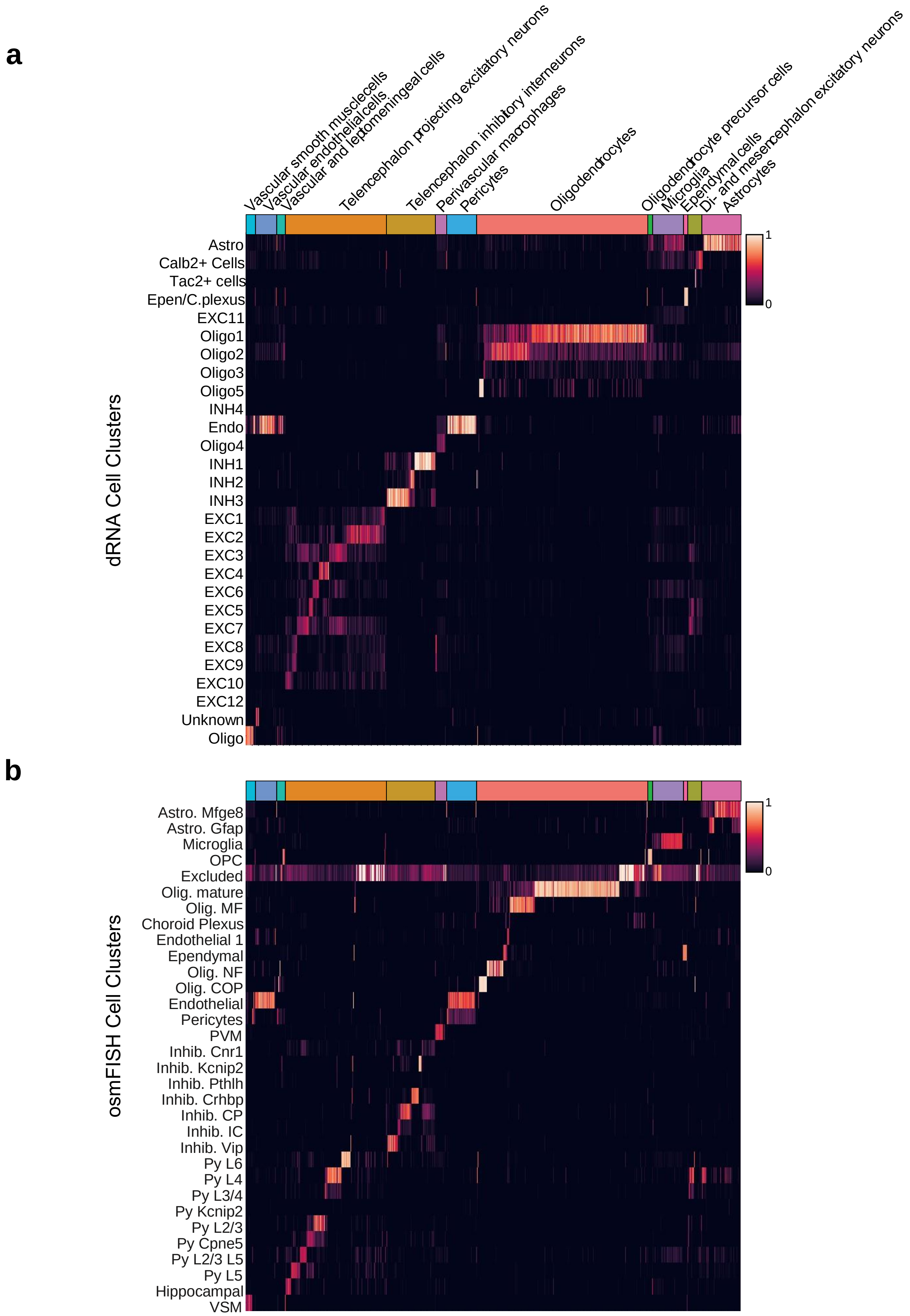

Supplementary Figure 9: Method comparison to single-cell RNA-sequencing defined clusters a, Comparison between the dRNA *de novo* clusters defined in Figure 2a and scRNA-seq clusters[1] when integrated using Spatial Gene Enrichment.

b, Comparison between the osmFISH clusters, described in Codeluppi *et al.*[3], and scRNA-seq clusters[1] when integrated using Spatial Gene Enrichment.

Supplementary References

1. Zeisel, A. et al. Molecular Architecture of the Mouse Nervous System. Cell 174, 999-1014.e22 (2018).
2. Lein, E. S. et al. Genome-wide atlas of gene expression in the adult mouse brain. Nature 445, 168–76 (2007).
3. Codeluppi, S. et al. Spatial organization of the somatosensory cortex revealed by osmFISH. Nat. Methods 15, 932–935 (2018).
